# Computing evolutionary distinctiveness indices in large scale analysis

**DOI:** 10.1186/1748-7188-7-6

**Published:** 2012-04-13

**Authors:** Iain Martyn, Tyler S Kuhn, Arne O Mooers, Vincent Moulton, Andreas Spillner

**Affiliations:** 1 IRMACS and BioSciences, Simon Fraser University, 8888 University Drive, Burnaby, V5A 1S6 Canada; 2 Department of Biology, The Pennsylvania State University, 208 Mueller Laboratory, University Park, PA USA 16802; 3 School of Computing Sciences, University of East Anglia, Norwich NR4 7TJ UK; 4 Department of Mathematics and Computer Science, University of Greifswald, Germany

## Abstract

We present optimal linear time algorithms for computing the Shapley values and 'heightened evolutionary distinctiveness' (HED) scores for the set of taxa in a phylogenetic tree. We demonstrate the efficiency of these new algorithms by applying them to a set of 10,000 reasonable 5139-species mammal trees. This is the first time these indices have been computed on such a large taxon and we contrast our finding with an ad-hoc index for mammals, fair proportion (FP), used by the Zoological Society of London's EDGE programme. Our empirical results follow expectations. In particular, the Shapley values are very strongly correlated with the FP scores, but provide a higher weight to the few monotremes that comprise the sister to all other mammals. We also find that the HED score, which measures a species' unique contribution to future subsets as function of the probability that close relatives will go extinct, is very sensitive to the estimated probabilities. When they are low, HED scores are less than FP scores, and approach the simple measure of a species' age. Deviations (like the *Solendon *genus of the West Indies) occur when sister species are both at high risk of extinction and their clade roots deep in the tree. Conversely, when endangered species have higher probabilities of being lost, HED scores can be greater than FP scores and species like the African elephant *Loxondonta africana*, the two solendons and the thumbless bat *Furipterus horrens *can move up the rankings. We suggest that conservation attention be applied to such species that carry genetic responsibility for imperiled close relatives. We also briefly discuss extensions of Shapley values and HED scores that are possible with the algorithms presented here.

## 1 Introduction

A phylogenetic tree is a directed graph that portrays the evolutionary relationships among its leaves. The shape of a phylogenetic tree of species can also be viewed as a measure of the redundant and unique evolutionary information embodied in the species: a species in a large and recently-diverged genus like *Mus *shares much of its evolutionary history with many other species, while the monotypic platypus (*Ornithorhynchus anatinus*) embodies a large amount of mammalian evolutionary information not found elsewhere (as expressed in its peculiar genome [1] and phenotype [2]).

Importantly, many species across the tree of life are at risk of extinction due to human activity. Motivated by the need to objectively prioritize conservation effort in an age of triage [3], the Zoological Society of London (ZSL) is spearheading a conservation campaign that identifies those species that are at once imperiled with extinction and that are minimally redundant within their taxonomic group (http://www.edgeofexistence.org). There are many measures of this evolutionary redundancy [4], but all have the common feature that species with fewer closer relatives are given higher rank. The measure chosen by the ZSL is called 'fair proportion' (FP), and is a weighted sum of the edge lengths along the path from the root of an ultra-metric tree to a leaf, with the weights being 1/number of species that share that edge [5]. FP has the useful property that the sum of the values across the species is the sum of all the edgelengths of the tree (this sum of edgelengths of a (sub)tree is often called the Phylogenetic Diversity (PD) of the tree [6]). So, across all mammals, the platypus has the highest FP score. Under the EDGE approach, FP scores are then multiplied by the probability of extinction for a species to produce an 'EDGE' score (for Evolutionarily Distinct and Globally Endangered), allowing for a global ranking of species within a higher taxon to help in the allocation of conservation attention.

Interestingly, FP scores are very highly correlated across simulated trees to the Shapley values [7], which is the expected increase in PD that a focal species brings to unrooted trees representing equiprobable subsets of taxa [8]. This measure was adapted by Steel and colleagues [9] to capture the extra PD a species brings to future unrooted subsets, where subsets are sampled in proportion to their probability of persisting in the future. It is called 'heightened evolutionary distinctiveness' (HED) and falls in a family of 'expected PD' measures [10]. The HED score correlates with the length of the pendant edge leading to the corresponding leaf of the tree (see below), but also highlights species that will become increasingly distinctive if and when imperiled relatives go extinct.

Collen et al. recently [11] published an updated ranking of mammals based on the FP score, based on 1,000 reasonable 5020-tip mammal trees. The authors suggested that the HED scores would be interesting to compare to the FP scores, but given that the fastest previously known algorithm for computing HED scores [8] has a quadratic run time, they did not compute these scores. Here, we first introduce fast (linear-time) algorithms for computing both Shapley values and HED scores, and then apply and compare these scores with FP scores across an improved sample of 10,000 near-complete (5139 species) mammal trees.

## 2 Methods

Let T=(V,E,λ) be an unrooted, edge-weighted phylogenetic tree on a set *X *with *n *taxa. Here *V *and *E *denote the set of vertices and edges of the tree and *λ *is a map that assigns to every edge *e *∈ *E *a non-negative real number, the *length λ*(*e*) of this edge. With every edge *e *of  T  is associated a *split S*_*e *_of *X*. For any *x *∈ *X*, we denote by *S*_*e*_(*x*) that set in *S*_*e *_that contains *x *and by ${\bar{S}}_{e}\left(x\right)$ the other set. In addition, for any subset $Y\subseteq X,PD_{T}\left(Y\right)$ denotes the total length of the smallest subtree of  T  containing the taxa in *Y*, also known as the *phylogenetic diversity *of *Y *with respect to  T  (see e.g. [6]). In the following we first define the two indices we will focus on in this paper and then present optimal linear time algorithms for computing them.

### 2.1 The Shapley value

In [8] the Shapley value $\psi_{x}^{sh}\left(T\right)$ of a taxon *x *∈ *X *with respect to an unrooted, edge-weighted phylogenetic tree T=(V,E,λ) is defined as follows:

$$\begin{array}{llll} \psi_{x}^{sh}\left(T\right) & =\frac{1}{n!}\underset{Z\subseteq X,x\in Z}{\sum }\left(|Z|-1\right)!\left(\left|X|-|Z\right|\right)!\; & & \;\\ & \cdot \left(PD_{T}\left(Z\right)-PD_{T}\left(Z-\left\{x\right\}\right)\right)\; & & \;\\ & \; & \end{array}$$

In [8] it is also shown that the Shapley value of a taxon with respect to a phylogenetic tree  T  is a certain linear combination of the lengths of the edges of  T . More specifically, fixing any taxon *x*, we have:

$$\psi_{x}^{sh}\left(T\right)=\underset{e\in E}{\sum }\frac{\left|{\bar{S}}_{e}\left(x\right)\right|}{\left|X||S_{e}\left(x\right)\right|}\lambda \left(e\right)$$

Note that the fact that the coefficients in this linear combination can be computed in polynomial time implies that the Shapley value of *x *with respect to  T  can be computed in polynomial time. In fact, an algorithm with run time *O*(*n*^2^) is presented in [8]. An implementation of this algorithm is available as part of the Bio::Phylo software package [12].

### 2.2 Heightened evolutionary distinctiveness (HED)

In [9] the index HED was introduced which is defined as follows. Let *p *: *X *→ [0, 1] be a map that assigns to each *x *∈ *X *a real number in the closed interval between 0 and 1. We can interpret *p*(*x*) as the probability that taxon *x *will go extinct within a certain amount of time in the future. Then, for any *x *∈ *X*, the HED of *x *with respect to an unrooted, edge-weighted phylogenetic tree T=(V,E,λ) is defined as follows:

$$\begin{array}{r} \psi_{x}^{hed}(T)=\sum_{Z\subseteq X-\{x\}}((\prod_{y\in Z}(1-p(y))\prod_{y\in X-(Z\cup \{x\})}p(y))\\ \cdot (PD_{T}(Z\cup \{x\})-PD_{T}(Z))) \end{array}$$

It is shown in [9] that, similarly to the Shapley value, the HED index is a linear combination of the lengths of the edges of  T , namely:

$$\begin{array}{llll} \psi_{x}^{hed}\left(T\right) & =\underset{e\in E}{\sum }\left(\underset{y\in \left(S_{e}\left(x\right)-\left\{x\right\}\right)}{\prod }p\left(y\right)\right)\cdot \; & & \;\\ & \left(1-\underset{y\in {\bar{S}}_{e}\left(x\right)}{\prod }p\left(y\right)\right)\cdot \lambda \left(e\right)\; & & \;\\ & \; & \end{array}$$

### 2.3 Linear time algorithms

**Figure 1:**
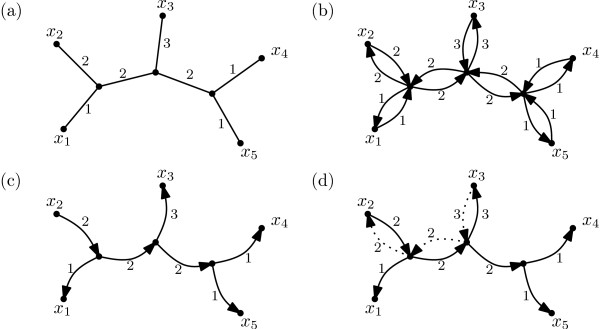
**(a) A phylogenetic tree on *X *= {*x*_1_, *x*_2_, . . ., *x*_5_}. **(b) Replacing each edge by a pair of directed arcs. (c) The arcs in the set $A_{x_{2}}$. (d) The change from $A_{x_{2}}$ to $A_{x_{3}}$.

The basic idea for the design of a linear time algorithm for computing the Shapley values and the HED indices of the taxa with respect to a phylogenetic tree  T  is very similar. It will be convenient to replace, in the given phylogenetic tree  T , every edge *e *= {*v*, *w*} by a pair of directed arcs (*v*, *w*) and (*w*, *v*) and put *λ*(*v*, *w*) = *λ*(*w*, *v*):= *λ*(*e*) (see Figure 1 for an example).

Let *A *denote the set of arcs we have introduced. For every *x *∈ *X*, we denote by *A*_*x *_the subset of those arcs in *A *that are directed away from *x *(see Figure 1(c)). Note that, for the Shapley value, the linear combination that yields $\psi_{x}^{sh}\left(T\right)$ for a taxon *x *∈ *X *can be expressed as follows: With each *a *∈ *A *is associated the real number

$$\kappa \left(a\right)=\frac{\left|{\bar{S}}_{e}\left(y\right)\right|}{\left|X||S_{e}\left(y\right)\right|}$$

where *e *is the edge that gave rise to arc *a *and *y *is an arbitrary element in *X *such that *a *is directed away from *y*. Then we have

$$\psi_{x}^{sh}\left(T\right)=\underset{a\in A_{x}}{\sum }\kappa \left(a\right)\cdot \lambda \left(a\right).$$

Now, fix an ordering *x*_1_, *x*_2_, . . ., *x*_*n *_of the taxa in *X *that corresponds to walking around a planar drawing of  T  (see Figure 1(a)). Assume we have already computed the value $\psi_{x_{i}}^{sh}\left(T\right)$ for some *i *∈{1, 2, . . ., *n *− 1}. Then it is not hard to see (cf. Figure 1(d)) that we have

$$\begin{array}{c} \psi_{x_{i+1}}^{sh}\left(T\right)=\psi_{x_{i}}^{sh}\left(T\right)-\left(\underset{a\in \left(A_{x_{i}}-A_{x_{i+1}}\right)}{\sum }\kappa \left(a\right)\cdot \lambda \left(a\right)\right)\\ +\left(\underset{a\in \left(A_{x_{i+1}}-A_{x_{i}}\right)}{\sum }\kappa \left(a\right)\cdot \lambda \left(a\right)\right), \end{array}$$

that is, it suffices to consider the arcs that correspond to the edges of  T  that lie on the unique path from *x*_*i *_to *x*_*i*+1_.

Our algorithm for computing the Shapley values for all *x *∈ *X *can be summarized as follows:

(1) Select a suitable ordering *x*_1_, *x*_2 _,. . ., *x*_*n *_of *X*.

(2) Compute, for each arc *a *∈ *A*, the value *κ*(*a*).

(3) Compute $\psi_{x_{1}}^{sh}\left(T\right)$.

(4) For *i *=1, 2, . . ., *n *− 1, compute $\psi_{x_{i+1}}^{sh}\left(T\right)$ from $\psi_{x_{i}}^{sh}\left(T\right)$.

It is not hard to see that, if  T  is given in Newick-format [13], steps (1)-(3) have a run time in *O*(*n*). To establish that also step (4) has a run time in *O*(*n*), it suffices to note that every edge of  T  is involved in the computation of $\psi_{x_{i+1}}^{sh}\left(T\right)$ from $\psi_{x_{i}}^{sh}\left(T\right)$ for at most two *i *∈{1, 2, . . ., *n *− 1}.

Now we turn to the HED index. In analogy to the definition of the values *κ*(·) above, we put

$$\kappa^{\prime }\left(a\right)=\left(\underset{y\in S_{e}\left(z\right)}{\prod }p\left(y\right)\right)\cdot \left(1-\underset{y\in {\bar{S}}_{e}\left(z\right)}{\prod }p\left(y\right)\right)$$

for each *a *∈ *A*, where *e *is the edge that gave rise to arc *a *and *z *is an arbitrary element in *X *such that *a *is directed away from *z*. Then, for each *x *∈ *X*, we clearly have

$$p\left(x\right)\cdot \psi_{x}^{hed}\left(T\right)=\underset{a\in A}{\sum }\kappa^{\prime }\left(a\right)\cdot \lambda \left(a\right).$$

So, in a preprocessing step we compute *κ′*(*a*) for all *a *∈ *A *in linear time. Then we can apply our algorithm above to compute $p\left(x\right)\cdot \psi_{x}^{hed}\left(T\right)$ for each *x *∈ *X*, simply replacing the values *κ*(*a*) by *κ′*(*a*) for all *a *∈ *A*. This immediately yields the values $\psi_{x}^{hed}\left(T\right)$ for those *x *∈ *X *with *p*(*x*) *>*0 in linear time.

It remains to describe how we can deal with those *x *∈ *X *with *p*(*x*) = 0. For a subset *A *⊆ *X*, let A* denote the set of those *x *∈ *A *with *p*(*x*) *>*0. Then we put

$${\tilde{\kappa }}^{\prime }(a)=\{\begin{array}{cc} \left(\prod_{y\in S_{e}^{*}}p(y)\right) & \text{if}|S_{e}^{*}(z)|\geq |S_{e}(z)|-1\\ \cdot (1-\prod_{y\in {\bar{S}}_{e}}p(y)\text{)} & \\ 0 & \text{otherwise} \end{array}$$

for each *a *∈ *A*, where, as before, *e *is the edge that gave rise to arc *a *and *z *is an arbitrary element in *X *such that *a *is directed away from *z*. In addition, define

$${\tilde{\psi }}_{x}\left(T\right)=\underset{a\in A}{\sum }{\tilde{\kappa }}^{\prime }\left(a\right)\cdot \lambda \left(a\right)$$

for every *x *∈ *X*. It is not hard to check that ${\tilde{\psi }}_{x}(T)=\psi_{x}^{hed}(T)$ holds for all *x *∈ *X *with *p*(*x*) = 0 and, therefore, also these values can be computed in linear time.

## 3 Application

We tested the utility of the new linear time algorithms for the Shapley values and HED scores by applying them to an updated version of the complete mammal tree the ZSL used to generate EDGE scores [11]. We outline the dataset and implementation below.

### 3.1 Dataset

One issue with producing distinctiveness indices is how tree uncertainty is incorporated [14][11]. The current supertree of mammals is only 50% resolved [15], and the resulting polytomies produce edge lengths that are biased long - in other words, species in polytomies seem older and more distinctive than they should. Collen et al. dealt with this by producing a sample of 1000 trees that each resolve these polytomies via Bayesian methods outlined in Kuhn et al. [15]. To obtain our dataset, we followed this method (given in further detail in the next section) and increased the sample size to 10,000 trees to obtain a better picture of the uncertainty.

A further and more vexing issue with producing evolutionary redundancy indices, especially for large trees, is dealing with taxonomic instability. The current mammal species supertree contains 5020 species [16]. Collen et al. found 396 species from the third and most recent edition of 'Mammal Species of the World' (MSW3) [17] that were not found on this tree (producing an interim taxonomic list of 5416 species). Of these 396 species, however, 75 are well-known to be extinct [18], leaving only 321 problem species.

Collen et al. estimated fair proportion scores for 250 of these remaining species by attributing to each the average of the scores across its relevant genus. For two further species of conservation concern (*Pseudoryx nghetinhensis *and *Laonastes aenigmamus*), molecular estimates of likely time of divergence were used to construct estimates of FP scores. We conducted a literature search and were able to estimate locations for all these 252 species by placing them next to their presumed sister taxon and dividing the edge length in half.

Finally, we needed to deal with taxonomic conflict between the reconciled MSW3 + supertree taxonomy and the International Union for Conservation (IUCN) taxonomy from which we drew information on imperilment. Collen et al. found that the IUCN lists a further 28 species in the MSW3 taxonomy as extinct, as well as reclassifying 31 as subspecies, and demonstrating that 74 are synonyms for other species already on the supertree. Whereas Collen et al. simply removed all these tips from their final ranking after estimating fair proportion scores, we dropped these taxa from our trees first, leaving a total of 5139 mammal species that reconciles the MSW3 taxonomy with the latest IUCN taxonomic notes, and which leaves 1 percent ((321-252)/5208) mammal species as 'pseudo-extinct'. In passing, we call for a consortium to organize a mammal database to offer a stable single source for taxonomic and biological information for this and other important taxa.

### 3.2 Implementation

The algorithms were implemented in R v2.12.0 [19] and fashioned so as to read in trees in the Newick format, one of the most common and simplest formats for storing trees [13]. Prior to application the algorithms were tested on different size randomly generated Yule trees and it was confirmed that they ran in linear time (data not shown). All analyses were carried out on a single core of a Quad Core Intel Xeon 2.33 Ghz processor with 16 GB of memory. The R-script is available upon request.

Trees were produced according to the method described in Kuhn et al. [15] with one further modification: uncertainty in individual node ages on the mammal supertree was accomodated by allowing for each node age to be drawn from a prior constrained normal distribution (see also [11]). The mean of each distribution was the best age estimate given by Fritz et al. [16] and each corresponding standard deviation was simply given as (best-worst estimate)/1.96, where the worst estimate is the estimate furthest from the best. The resolved trees were produced using BEAST v.1.6.2 [20]. The set of 10,000 trees represent a combined output from 8 independent runs. For each run trees were recorded every 2,000 steps after a burn-in period of at least 400,000. The burn-in was chosen based on visual examination of log files in Tracer v1.5 [21].

We assigned probability of extinction values to each of the remaining taxa based on the most recent threat level assessment by the IUCN, following the method of Mooers et al. [22]. Briefly, each of the five indicative IUCN categories (critically endangered, endangered, vulnerable, near threatened, and least concern) is assigned a discrete value. However, since there is as of yet no definitive set of values to use, we used two: the 'Isaac' set where the values assigned to categories are 0.4, 0.2, 0.1, 0.05, 0.025 respectively (so, a doubling of risk with each increase in imperilment rank, and the 'IUCN 100' set where values are 0.999, 0.667, 0.1, 0.01, 0.0001 respectively. For the 696 data deficient species that could not be placed into one of the five categories, we assigned the weighted mean of the probability of extinction vlaues of the the other species. For the first set this was 0.07 and for the second was 0.11. Thus for the first set we performed two runs, one equating data deficient with near threatened status (probability of extinction = 0.05) and one equating data deficient with vulnerable status (probability of extinction = 0.1). These two analyses were then combined to yield a more realistic final result. For the latter set we could equate data deficient as vulnerable (probability of extinction = 0.1).

## 4 Results and Discussion

### 4.1 FP scores vs Shapley values

**Figure 2:**
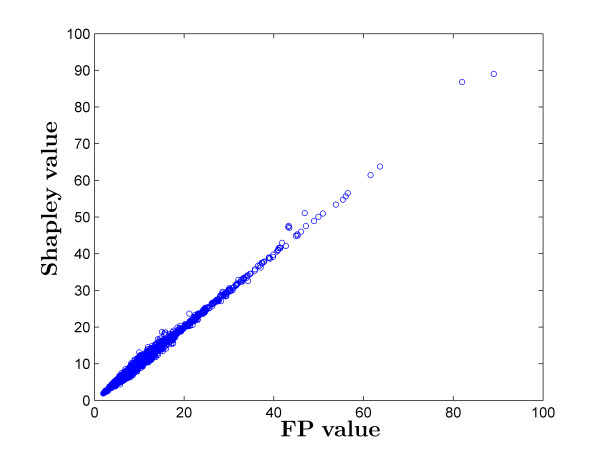
**Shapley values vs. FP scores for all mammal species (r =0.9998)**.

In Additional File 1 we report FP scores, Shapley values, and HED scores for all 5139 species, where the scores are the average over the set of 10,000 trees. As we expected, Shapley values and FP scores are very strongly correlated (Figure 2). As proven by Hartmann [7], the Shapley value for a given taxa should approach the FP score as the number of elements in *X *tends to infinity. For full details the reader is directed to Hartmann, but this result may be intuited (viewing some interior vertex *r *of the given tree  T  as a root) by breaking down the Shapley value as

$$\begin{array}{c} \psi_{x}^{sh}\left(T\right)=\underset{e\in s\left(T,x,r\right)}{\sum }\frac{\left|{\bar{S}}_{e}\left(x\right)\right|}{\left|X||S_{e}\left(x\right)\right|}\lambda \left(e\right)\\ +\underset{e\notin s\left(T,x,r\right)}{\sum }\frac{\left|{\bar{S}}_{e}\left(x\right)\right|}{\left|X||S_{e}\left(x\right)\right|}\lambda \left(e\right) \end{array}$$

and noting that the FP score is defined as

$$\psi_{x}^{fp}\left(T\right)=\underset{e\in s\left(T,x,r\right)}{\sum }\frac{\lambda \left(e\right)}{\left|S_{e}\left(x\right)\right|}$$

where s(T,x,r) denotes the set of edges forming the path in  T  from *x *to the root *r*. Consider the 5 outliers (*Zaglossus attenboroughi, Zaglossus bartoni, Zaglossus bruijni, Tachyglossus aculeatus*, and *Ornithorhynchus anatinus*). These taxa form a monophyletic group (Order Monotremata) directly connected to the root of the tree. They thus receive higher Shapley values than FP scores because while the left term in the Shapley value approaches the FP term, the right term, especially for deep connecting branches between this group and the rest of the tree, cannot be approximated as zero. As a result, they gain an additional significant positive contribution to their Shapley value that is not incorporated in their FP value. Another way to intuit this result is to note that the Shapley value is for unrooted trees, and so the additional PD that any monotreme contributes to a possible future subset includes both its distance to the root and the stem age of the placental mammals.

### 4.2 FP scores vs HED scores

**Figure 3:**
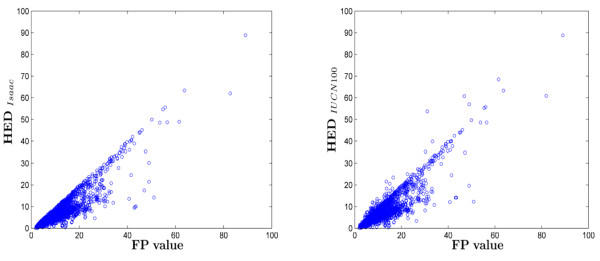
**Comparison between HED and FP values for all mammal taxa**. The left HED was calculated using the 'Isaac' probability of extinction scores (r = 0.8984) and the right was calculated using 'IUCN 100' probability of extinction scores (r = 0.8869).

Due to the similarity between Shapley values and FP scores, we focussed on comparing HED with FP. As observed in Figure 3, HED generated with both sets of probability of extinction estimates also correlate well with FP (r = 0.8984 for 'Isaac', r = 0.8869 for IUCN 100) though there remains significant scatter. We explain this behaviour by noting that HED correlates even more strongly with just the pendant edge of each tip (r = 0.9947, r = 0.9507), suggesting that the internal tree structure is less relevant. This makes sense, as while for Shapley and FP the contribution for deeper edges can be seen to be approximately proportional to 1/n, for HED the contribution is approximately proportional to *a^n^*, where most often *a <<*1. This of course is a much faster decrease and effectively reduces all edges below the pendant edges to higher order negligible terms. We observe as well that for the 'Isaac' set of extinction values it is impossible for these higher order terms to be equal or greater to the equilavent terms for Shapley values and FP scores, even if every taxa on the tree was critically engangered (0.4).

As suggested by Faith [10] this exclusion of deeper edges can oftentimes be justified, as Shapley values and FP scores can overestimate the contribution of an interior branch. For example, an imperiled species belonging to a relatively recent diverging clade with a long interior edge will score highly despite the fact that perhaps its sister taxa are in no danger of extinction. In this case the evolutionary distinctiveness represented by the long interior edge is in little danger of being lost even if the more endangered species go extinct. This is manifest for *Ornithorhynchus anatinus*, a species we encoutered earlier as one of the furthest outliers in Figure 2. The platypus, as its more commonly known, is the oldest member of *Monotremata *and is sister to the Echnidas. While the remaining species are critically endangered, the short-faced echidna *Tachyglossus aculeatus *is listed by the IUCN as least concern. This is enough to, in Faith's words, "secure the interior branch" and thus lower the ranking of any species between *Tachyglossus aculeatus *and the interior branch (here just *Ornithorhynchus anatinus*). Similar situations where a sister species or down-tree species is relatively safe explain the majority of divergence between FP and HED.

Figure 3 also illustrates the effect of changing the probabilites of extinction. When some species are at very high risks of extinction, HED can be greater than FP, because they are expected to contribute large amounts to the tree following extinction of close relatives. This is most intuitively seen with the two remaining elephant species. While it is inconceivable that the Indian elephant *Elephas maximus *would be allowed to go extinct (as it breeds in zoos), the relatively less imperiled African elephant *Loxodonta africana *carries genetic responsibility for its close cousin. The two solendons of the West Indies offer another example. While on remarkably old pendant edges (40 my), they also jointly root very deep in the tree (at 82 mya). Because both are critically endangered, it is not unlikely that one will end up being the sole representative of their (now) shared interior branch.

These examples suggest it may be profitable to take a more dynamic view of how individual species represent the evolutionary history of their group. The fair proportion metric as used by the EDGE programme is an intuitively compelling measure of evolutionary distinctiveness, and, at the limit, it approaches the well-characterized Shapley value. It may be that considering all future subsets of taxa to be equally likely is a conservative approach to measuring worth (the future is indeed grim for much of biodiversity, and projections based on current imperilment are very imprecise). However, as argued forcefully by Faith [10], it may also make sense to consider future expected PD more explicitly, such that an HED-style metric should be considered. We suggest that, at the very least, some attention be given to species that are relatively cheap to manage (because they are not yet in grave danger, such as the short-faced echidna) and that are also expected to represent large swathes of biodiversity under worst-case scenarios (e.g. if we were to lose all currently-imperiled monotremes).

## 5 Extensions

The fact that the Shapley and HED values are measures of evolutionary distinctiveness on unrooted trees suggests that the above approach to highlighting imperiled and evolutionarily isolated bits of biodiversity could be extended from species on a tree to populations connected via a network on the landscape. Importantly, the algorithms presented here for computing Shapley values and HED scores lend themselves naturally to split networks [23]. The motivation for such an extension comes from the observation that prioritizing populations within species may present policymakers with a useful tool after a species has been legally listed (e.g. through an Endangered Species Act) for conservation management. Once a species has been awarded protection, and funds are allocated for survival and recovery, an early step in any management plan is to assess how many populations there are, what state each is in, how they are demographically and genetically connected on the landscape, and where genetic diversity lies. As when arguing for a triage approach to species conservation, it may be useful and efficient to highlight those populations of an endangered species that are at once distinctive and that carry genetic responsibility for other populations. Costs and benefits may be easier to compare within than between species, such that objective decisions as to where to invest scarce conservation resources may be more palatable.

## 6 Competing interests

The authors declare that they have no competing interests

## 7 Authors' contributions

AS, VM conceived of the algorithm, AS produced the equations, TK created the trees, and IM and AM implemeted the algorithm, perfomed the study, and wrote the first draft of the paper. All authors read and approved the final manuscript.

## Supplementary Material

Additional File 1 **Complete Scores**. This file contains the average Shapley, HED, and FP scores for all 5139 mammal species across the 10,000 tree distribution, as well the respective standard deviations on this average. It also contains the IUCN threat category for each species at the time of writing. Click here for file
